# Association of thrombus density and endovascular treatment outcomes in patients with acute ischemic stroke due to M1 occlusions

**DOI:** 10.1007/s00234-022-02971-4

**Published:** 2022-05-16

**Authors:** Agnetha A. E. Bruggeman, Nyk Aberson, Manon Kappelhof, Bruna G. Dutra, Jan W. Hoving, Josje Brouwer, Manon L. Tolhuisen, Nerea Arrarte Terreros, Praneeta R. Konduri, Nikki Boodt, Yvo B. W. E. M. Roos, Wim H. van Zwam, Reinoud Bokkers, Jasper Martens, Henk A. Marquering, Bart J. Emmer, Charles B. L. M. Majoie, the MR CLEAN Registry investigators

**Affiliations:** 1 Department of Radiology and Nuclear Medicine, Amsterdam University Medical Centers, University of Amsterdam, Room G1-240, Meibergdreef 9 1105 AZ, Amsterdam, The Netherlands; 2 Department of Biomedical Engineering and Physics, Amsterdam University Medical Centers, University of Amsterdam, Amsterdam, The Netherlands; 3 Department of Neurology, Amsterdam University Medical Centers, University of Amsterdam, Amsterdam, The Netherlands; 4 Department of Radiology and Nuclear Medicine, Erasmus University Medical Center, Rotterdam, The Netherlands; 5 Department of Neurology, Erasmus University Medical Center, Rotterdam, The Netherlands; 6 Department of Radiology and Nuclear Medicine, Cardiovascular Research Institute Maastricht (CARIM), Maastricht University Medical Center, Maastricht, The Netherlands; 7 Department of Radiology and Nuclear Medicine, University Medical Center Groningen, Groningen, The Netherlands; 8 Department of Radiology and Nuclear Medicine, Rijnstate Hospital, Arnhem, The Netherlands

**Keywords:** Stroke, Thrombectomy, Thrombus density, Reperfusion

## Abstract

**Purpose:**

We aimed to study the association of non-contrast CT (NCCT) thrombus density with procedural and clinical outcomes in patients with acute ischemic stroke who underwent endovascular treatment (EVT). Since thrombus density is associated with thrombus location, we focused on M1 occlusions only.

**Methods:**

Patients with available thin-slice (< 2.5 mm) NCCT were included from a nationwide registry. Regression models were used to assess the relation between thrombus density (per Hounsfield unit [HU]) and the following outcomes. For reperfusion grade, adjusted common odds ratios (acOR) indicated a 1-step shift towards improved outcome per HU increase in thrombus density. For the binary outcomes of first-pass reperfusion (first-pass extended thrombolysis in cerebral infarction [eTICI] 2C-3, FPR), functional independence [90-day modified Rankin Scale (mRS) score of 0–2] and mortality), aORs were reported. Adjusted *β* coefficients (a*β*) were reported for 24-h NIHSS and procedure duration in minutes. Outcome differences between first-line treatment devices (stent retriever versus aspiration) were assessed with interaction terms.

**Results:**

In 566 patients with M1 occlusions, thrombus density was not associated with reperfusion (acOR 1.01, 95% CI 0.99–1.02), FPR (aOR 1.01, 95% CI 0.99–1.03), mortality (aOR 0.98, 95% CI 0.95–1.00), 24-h NIHSS (a*β* − 0.7%, 95% CI − 1.4–0.2), or procedure duration (a*β* 0.27, 95% CI − 0.05–0.58). In multivariable analysis, thrombus density was associated with functional independence (aOR 1.02, 95% CI 1.00–1.05). No interaction was found between thrombus density and first-line treatment device for any outcome.

**Conclusion:**

In patients with M1 occlusions, thrombus density was not clearly associated with procedural and clinical outcomes after EVT.

**Supplementary Information:**

The online version contains supplementary material available at 10.1007/s00234-022-02971-4.

## Introduction

Endovascular treatment (EVT) for acute ischemic stroke is now standard of care for patients with a proximal occlusion of the anterior circulation [1]. The main goal of EVT is to retrieve the thrombus to restore the blood flow to the brain. Nevertheless, successful reperfusion is not achieved in nearly 20% of EVT-treated patients in current clinical practice [2]. Thrombus imaging characteristics could potentially guide in choosing EVT technique (e.g., stent retriever or distal aspiration) [3]. Previously, thrombus density has been reported to be associated with reperfusion in patients who underwent EVT [4–7]. However, other studies found no association between thrombus density and reperfusion and functional outcome in patients treated with EVT [8–12].

Histologically, hyperdense thrombi on non-contrast CT (NCCT) contain more red blood cells (RBCs) than non-hyperdense thrombi which are more fibrin- and platelet-rich [3, 13–17]. Fibrin-rich thrombi have been described to be stiffer and can cause more friction between the thrombus and vessel wall compared with RBC-rich thrombi. Consequently, fibrin-rich thrombi have been found to be more difficult to retrieve with EVT [3, 18]. This might explain previously reported associations between decreasing thrombus density, in Hounsfield units (HU), and a lower chance of successful reperfusion and favorable outcome [3–6]. Furthermore, RBC-rich thrombi are more sensitive to intravenous alteplase (IVT) than fibrin-rich thrombi [3]. There is heterogeneity in thrombus location in most studies investigating imaging characteristics of thrombi. Yet, hyperdense thrombi usually have a more proximal location (smaller distance from the terminus of the carotid artery [ICA-T] to proximal thrombus border) than non-hyperdense thrombi [11, 19]. Hence, this difference might affect the association between thrombus density and outcomes and could be an explanation for the conflicting results in previous studies.

We aimed to study the association between thrombus density, reperfusion, first-pass reperfusion, procedure duration, functional outcome, and mortality in a large dataset consisting of patients with occlusions of the M1 segment of the middle cerebral artery treated with EVT. We specifically focused on M1 occlusions to reduce the influence of other factors, such as vascular anatomy, clinical deficit, and procedural difficulty. Furthermore, we assessed the effect of first-line EVT device (stent retriever or aspiration) on the association between thrombus density and outcomes.

## Methods

### Patient selection

Patients included in this study were recruited from the MR CLEAN Registry: a nationwide prospective, observational multicenter registry that collected data of patients treated with EVT for acute ischemic stroke due to intracranial large vessel occlusion in the 17 intervention hospitals in the Netherlands, since the completion of the MR CLEAN trial in March 2014 [20]. The central medical ethics committee of the Erasmus MC gave permission to carry out the study as a registry (MEC-2014–235) [20]. Source data of this study are not available due to privacy regulations, but analytic methods and statistical code are available upon reasonable request.

The current study reports on patients treated between March 14, 2014 and November 1, 2017. All patients without contraindications received 0.9 mg/kg IVT prior to EVT. The exact EVT approach and material choice were left to the discretion of the treating neurointerventionist. EVT consisted of stent retriever thrombectomy, aspiration thrombectomy, or a combined approach, with or without administering additional intra-arterial thrombolytic agents. A combined approach of aspiration and stent retriever was not recorded separately. Therefore, these patients were included in the first-line stent retriever group. For the current study, we used the following inclusion criteria: occlusion of the M1 segment of the middle cerebral artery, age ≥ 18 years, onset to groin puncture < 6.5 h, treatment performed in a MR CLEAN trial center, and available thin-slice baseline NCCT and CT angiography (CTA) scans acquired within 30 min on the same scanner. For patients who were transferred from a primary stroke center, we used the primary stroke center’s imaging for thrombus density measurements. We excluded patients with a high chance of inaccurate measurements due to incorrigible co-registration misalignment, poor CTA contrast opacification, artifacts (movement, metal, beam hardening), excessive noise, too short or too narrow thrombi, or a thrombus located too close to bone causing bone artefacts, partial occlusions, bilateral thrombi, and incomplete field of views. Furthermore, we excluded patients with calcified cerebral emboli since the higher density values in these thrombi cause streak artefacts and partial volume artefacts. In addition, as we reported before [21], patients with calcified cerebral emboli have worse (reperfusion) outcomes than other stroke patients which could interfere with our analysis.

### Imaging analyses

The following imaging characteristics were evaluated by the MR CLEAN Registry imaging core lab, whose members were blinded to all clinical data except for symptom side [20]: Alberta Stroke Program Early CT Score (ASPECTS) on baseline NCCT [22], clot burden score [23, 24], collateral score [24], presence of cervical carotid lesions, and occlusion location on baseline CTA. Ipsilateral cervical carotid lesions were classified into non-significant or significant atherosclerotic stenosis (< or > 50%), total atherosclerotic occlusion (100%), or dissection. Reperfusion status, occurrence of per-procedural embolization to a new territory (ENT), and vessel perforation were evaluated by the imaging core lab on digital subtraction angiography imaging. Reperfusion status was evaluated according to the six-point expanded thrombolysis in cerebral infarction (eTICI) scale [25]. eTICI 0 or 1 indicates no or minimal reperfusion, eTICI 2 indicates incomplete reperfusion (2A < 50% of territory; 2B ≥ 50% of territory, 2C near complete reperfusion except slow flow or a few small distal cortical emboli), and eTICI 3 indicates complete reperfusion. For the current study, thin slice NCCT and CTA images (≤ 2.5 mm) were aligned with rigid co-registration using Elastix [26]. CTA images were used as reference for vessel anatomy and contrast detection. Thrombus density in Hounsfield units (HUs) was measured by placing three spherical ROIs with a 1-mm radius in the proximal, middle, and distal parts of the thrombus on co-registered NCCT and CTA images. Thrombus density was determined as the average density of the three NCCT ROIs in HU. Distance from ICA-T to proximal thrombus border (distance to thrombus [DT]) was measured as well to investigate the effect of thrombus location on thrombus density. DT was measured by placing one ROI in the ICA-T and one ROI at the proximal thrombus border and extracting the distance in mm. If necessary, additional ROIs were placed in between, to edit the used centerline in case of a curved artery. Measurements were performed by ten trained raters (AAEB, NA, MK, BGD, JWH, JB, MLT, NAT, PRK, NB). Regular consensus readings were held with an expert neuroradiologist with more than 25 years of experience (CBLMM) to discuss and compare measurements.

### Outcome assessment

Our primary outcome was final reperfusion grade as measured with the eTICI scale. Secondary outcomes were first-pass reperfusion (defined as excellent/complete reperfusion [eTICI 2C-3] in a single pass) [27], procedure duration (time between groin puncture and groin closure), 24-h National Institutes of Health Stroke Scale (NIHSS), 90-day modified Rankin Scale (mRS) [28, 29], and 90-day mortality. The mRS score was assessed at 90 days after stroke by local investigators as part of usual care. Functional independence was defined as an mRS score of 0–2.

### Statistical analysis

For descriptive analyses, we compared baseline and treatment variables in patients with hyperdense (≥ 50 HU) and non-hyperdense thrombi (< 50 HU). This threshold was selected based on the median thrombus density in our study and based on previous studies in which an affected middle cerebral artery had a density between 44 and 61 HU [30, 31]. We compared thrombus density across different scanner types to assess the effect of scanner type on thrombus density. Furthermore, we compared baseline characteristics and scanner types between our cohort of patients with M1 occlusions with available thin-slice imaging and the overall cohort of patients with M1 occlusions in the MR CLEAN Registry. Group comparisons were made using the Pearson chi-squared test or Mann–Whitney *U* test appropriate to the type of data. For the variable first-line EVT device, we made comparisons over the years since the use of an aspiration device became more common over the years. We compared the use of first-line stent retriever versus aspiration in patients with hyperdense and non-hyperdense thrombi included between 2014 and 2016 versus patients included in 2017.

We used univariable and multivariable regression analyses to assess the association between thrombus density (per HU) and outcomes. In all regression analyses, thrombus density was assessed as continuous variable per 1 HU. Ordinal regression was only performed if the proportional odds assumption for the ordinal variable was met. This assumption was met for our primary outcome (ordinal eTICI) but not for our secondary outcome measure 90-day mRS. Therefore, we assessed 90-day mRS as binary outcome (mRS 0–2 vs mRS 3–6). eTICI was assessed with ordinal logistic regression resulting in an (adjusted) common odds ratio ([a]cOR) and 95% confidence intervals (95% CI) for a 1-step shift towards better reperfusion outcome per HU increase in thrombus density. We used linear regression for continuous outcomes, resulting in (adjusted) beta coefficients ([a]*β*) with 95% CI. Twenty-four-hour NIHSS was log 10 transformed, to better meet the assumption of normally distributed residuals in linear regression, and we added 1 point to the NIHSS, so an original NIHSS score of 0 was equivalent to log 10 NIHSS + 1. The resulting (a)*β* coefficients indicate the percentage increase or decrease of the 24-h NIHSS per HU increase in thrombus density. For procedure duration, the (a)*β* coefficients indicate the duration increase in minutes per HU increase in thrombus density. For dichotomous outcomes (first-pass reperfusion, functional independence and mortality), we used binary logistic regression resulting in (adjusted) odds ratios ([a]OR) with 95% CI per HU increase in thrombus density. First-pass reperfusion was compared with both multiple pass reperfusion (eTICI 2C-3 in multiple passes) and no excellent reperfusion which was defined as eTICI < 2C independent of the number of passes.

To assess the effect of thrombus density on first-line treatment device, interaction between thrombus density and first-line treatment device (first-line stent retriever versus first-line aspiration) was tested for all outcomes in separate models by adding an interaction term (first-line treatment device × thrombus density). In case of non-significant interaction, subgroup analyses were exploratory.

Based on baseline imbalances and pre-specified prognostic factors, we adjusted for age, sex, baseline NIHSS, time from onset to first CT imaging time, IVT, DT, clot burden score, ASPECTS, and > 50% stenosis of the ipsilateral carotid artery. For the regression analyses only, missing data were imputed using multiple imputation based on relevant covariates and outcomes [32]. *p* values < 0.05 were considered statistically significant. All statistical analyses were performed with IBM SPSS Statistics 28.0.

## Results

In total, 566 patients with M1 occlusions were included in the analyses (Supplemental Fig. I). A major exclusion reason was the unavailability of thin-slice imaging. Patients with hyperdense thrombi were more often male than patients with non-hyperdense thrombi (56% vs 45%, *p* < 0.01) (Table 1). Baseline NIHSS was higher in patients with hyperdense thrombi than in patients with non-hyperdense thrombi (median 16, IQR 13–20 vs 15, IQR 10–20; *p* = 0.006). Although the median ASPECTS and clot burden score were numerically similar between the two groups of patients, a significant difference was present (*p* = 0.005 and *p* = 0.02, respectively). Overall, median thrombus density was 50 HU (IQR, 44–57 HU). Thrombus density was similar across different scanner types (*p* = 0.08) (Supplemental Table I). Hyperdense thrombi had smaller DT than non-hyperdense thrombi: median 10 mm (IQR, 4–16 mm) vs 14 mm (IQR, 9–19 mm) (*p* < 0.001). High-grade stenosis of the ipsilateral carotid artery was more often present in patients with hyperdense thrombi vs non-hyperdense (*p* = 0.02). Time from onset to first CT imaging was significantly longer in patients with hyperdense thrombi than in patients with non-hyperdense thrombi (69 min, IQR 52–116 min vs 81 min, IQR 55–125 min; *p* = 0.047). Stent retrievers were more often used as first-line EVT device in patients with hyperdense than in patients with non-hyperdense thrombi: 182/233 (78%) vs 140/214 (65%) (*p* = 0.003) (Table 2). Overall, the use of aspiration increased over the years (*p* < 0.001; Supplemental Table II). In addition, we only found a difference in first-line EVT device in patients with hyperdense versus non-hyperdense thrombi included after 2016 (*p* = 0.01; Supplemental Table III). Occurrence of ENT was similar in both groups: 8/252 (3%) in patients with hyperdense thrombi versus 8/239 (3%) in patients with non-hyperdense thrombi (Table 2).

**Table 1:** Baseline characteristics

Baseline characteristics	All patients (*n* = 566)	Hyperdense thrombi (*n* = 299)	Non-hyperdense thrombi (*n* = 267)	*p* value
Age, median (IQR), total *n*	72 (62–80), 566	73 (63–80)	71 (59–81)	0.51
Male, *n*/total *n* (%)	285/566 (50)	166/299 (56)	119/267 (45)	**0.009**
Medical history, *n*/total *n* (%)				
Diabetes	87/560 (16)	41/297 (14)	46/263 (18)	0.23
Hypertension	252/552 (46)	136/295 (46)	116/257 (45)	0.82
Hypercholesterolemia	137/540 (25)	80/286 (28)	57/254 (22)	0.14
Previous stroke	93/561 (17)	46/297 (16)	47/264 (18)	0.46
Myocardial infarction	75/555 (14)	41/297 (14)	34/258 (13)	0.83
Atrial fibrillation	145/557 (26)	75/295 (25)	70/262 (27)	0.73
Anticoagulation use (vitamin K antagonists)	79/562 (14	36/297 (12)	43/265 (16)	0.16
Direct anticoagulants	24/560 (4)	15/297 (5)	9/263 (3)	0.34
Clinical presentation				
Baseline NIHSS, median (IQR), total *n*	16 (11–20), 559	16 (13–20), 298	15 (10–20), 261	**0.006**
Prestroke mRS, *n*/total *n* (%)				
0–2	486/556 (87)	262/294 (89)	224/262 (85)	0.20
≥ 3	70/556 (13)	32/294 (11)	38/262 (15)	
Imaging characteristics				
Baseline ASPECTS score, median (IQR), total *n*	9 (8–10), 566	9 (8–10), 299	9 (8–10), 267	**0.005**
Clot burden score, median (IQR), total *n*	6 (6–7), 447	6 (6–7), 235	6 (6–7), 212	**0.02**
Thrombus absolute density in HU, median (IQR), total *n*^†^	50 (44–57), 566	56 (53–61), 299	44 (40–47), 267	** < 0.001**
Distance from ICA-T to thrombus (DT) in mm, median (IQR), total *n*^†^	13 (6–18), 566	10 (4–16), 299	14 (9–19), 267	** < 0.001**
Collaterals, *n*/total *n* (%)				0.45
0. Absent collaterals	35/557 (6)	21/294 (7)	14/263 (5)	
1. ≤ 50% filling of the occluded territory	206/557 (37)	105/294 (36)	101/263 (38)	
2. > 50% and < 100% filling of the occluded territory	221/557 (40)	124/294 (42)	97/263 (37)	
3. 100% filling of the occluded territory	95/557 (17)	44/294 (15)	51/263 (19)	
Ipsilateral atherosclerotic carotid artery stenosis > 50%, *n*/total *n* (%)	43/506 (9)	30/267 (11)	13/239 (5)	**0.02**
Ipsilateral total atherosclerotic occlusion of carotid artery, *n*/total *n* (%)	39/506 (8)	23/267 (9)	16/239 (7)	0.42
Ipsilateral dissection of carotid artery, *n*/total *n* (%)	7/506 (1)	4/267 (2)	3/239 (1)	1.00
IVT prior to EVT, *n*/total *n* (%)	413/565 (73)	225/299 (75)	188/266 (71)	0.22
Onset to first CT imaging in min, median (IQR), total *n*^‡^	74 (54–120), 406	69 (52–116), 218	81 (55–125), 188	**0.047**
Transfer patient, *n*/total *n* (%)	212/565 (38)	109/299 (37)	103/266 (39)	0.58
Onset to groin time in min, median (IQR), total *n*	185 (138–245), 561	171 (136–235), 297	195 (139–250), 264	0.051

Categorical variables are presented as *n*/*N* and percentage. Continuous variables are presented as median (interquartile range [IQR]), total number. Transfer patient: patients who initially presented at a primary stroke center and were transferred to a comprehensive stroke center for EVT

*NIHSS* National Institutes of Health Stroke Scale, *mRS* modified Rankin Scale, *M1* M1 segment of the middle cerebral artery, *ASPECTS* Alberta Stroke Program Early CT Score, *ICA-T* terminus of internal carotid artery, *IVT* intravenous alteplase treatment**,** *EVT* endovascular treatment

*p values < 0.05 were considerd significant

^†^Thrombus measurements were performed on thin-slice non-contrast CT (NCCT) and CT angiography (CTA) imaging. Median slice thickness of NCCT was 1 mm (IQR, 0.9–1 mm), and median slice thickness of CTA was 0.75 mm (IQR, 0.6–1 mm)

^‡^First CT imaging refers to the admission imaging used for thrombus imaging characteristics

**Table 2:** Treatment characteristics

	All patients (*n* = 566)	Hyperdense thrombi (*n* = 299)	Non-hyperdense thrombi (*n* = 267)	*p* value
Number of passes, median (IQR), total *n*	2 (1–3), 434	2 (1–3), 230	2 (1–3), 204	0.45
Onset to reperfusion time, median (IQR), total *n*^†^	235 (185–305), 533	225 (181–302), 283	243 (189–307), 250	0.11
First-line EVT device, *n*/total *n* (%)				
Stent retriever	322/447 (72)	182/233 (78)	140//214 (65)	**0.003**
Aspiration device	125/447 (28)	51/233 (22)	74/214 (35)	
Final reperfusion (eTICI) score, *n*/total *n* (%)				0.25
0	86/555 (16)	43/293 (15)	43/262 (16)	
1	12/555 (2)	5/293 (2)	7/262 (3)	
2A	88/555 (16)	47/293 (16)	41/262 (16)	
2B	127/555 (23)	65/293 (22)	62/262 (24)	
2C	71/255 (13)	34/293 (12)	37/262 (14)	
3	171/555 (31)	99/293 (34)	72/262 (28)	
ENT	16/491 (3)	8/252 (3)	8/239 (3)	0.91

Categorical variables are presented as *n*/*N* and percentage. Continuous variables are presented as median (interquartile range [IQR]), total number. eTICI 0 or 1 indicates no or minimal reperfusion, eTICI 2 indicates incomplete reperfusion (2A < 50% of territory; 2B ≥ 50% of territory, 2C near complete reperfusion except slow flow or a few small distal cortical emboli), and eTICI 3 indicates complete reperfusion; ENT: per-procedural embolization to a new territory ^†^or last contrast bolus

*EVT* endovascular treatment, *eTICI* expanded thrombolysis in cerebral infarction

*p values < 0.05 were considerd significant

Significant differences were present between the cohort of patients with M1 occlusions included in this study and the overall cohort of patients with M1 occlusions included in the MR CLEAN Registry. Hypertension and hypercholesterolemia were more often present in the overall cohort of M1 patients than in our cohort: 942/1779 (53%) vs 252/552 (46%) (*p* < 0.001) for hypertension and 534/1730 (31%) vs 137/540 (25%) (*p* = 0.001) for hypercholesterolemia (Supplemental Table IV). A collateral score of 3 was more often present in the overall cohort of M1 patients (352/1766 [20%]) than in our cohort (95/557 [17%], *p* = 0.03). Onset to groin puncture time was significantly lower in our study cohort than in the overall cohort of M1 patients (median 185 vs 195 min, *p* < 0.001). This difference might be caused by the lower number of transferred patients in our study cohort when compared to the overall cohort (212/565 [38%] vs 1014/1814 [56%], *p* < 0.001). In addition, in our cohort of M1 occlusion patients, EVT was more often performed in a university hospital when compared to the overall cohort of M1 patients (66% vs 56%, *p* < 0.001) (Supplemental Table IV). Finally, imaging was performed on less different scanner types in our study cohort when compared to the overall cohort of patients with M1 occlusions (*p* < 0.001) (Supplemental Table V).

### Outcomes

**Fig. 1:**
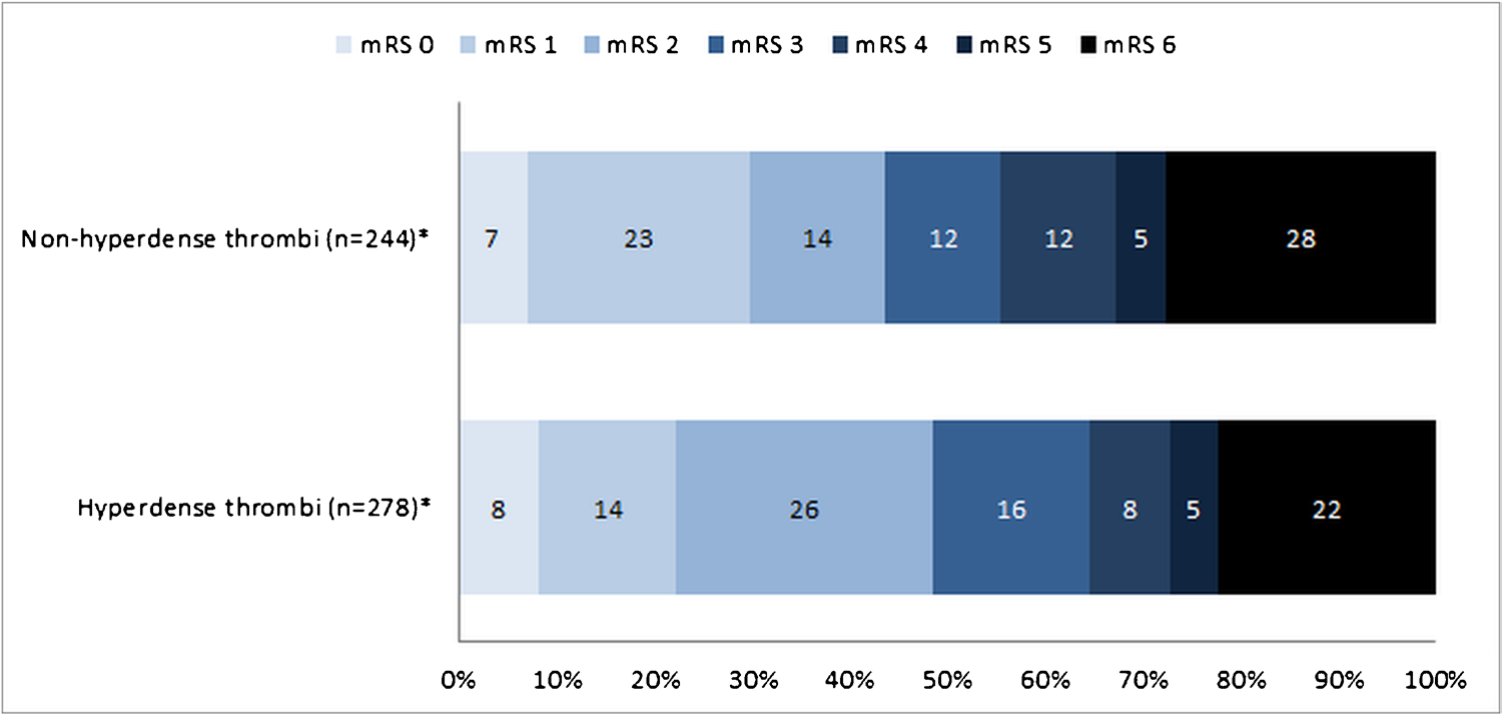
Ninety-day modified Rankin Scale (mRS) score in patients with non-hyperdense and hyperdense thrombi. Asterisk indicates the number of patients with available mRS score (*n* = 522, in 44 patients 90-day mRS was missing, these missing values were imputed for regression analyses only)

Thrombus density was not significantly associated with final reperfusion grade (acOR 1.01, 95% CI 0.99–1.02) or first-pass reperfusion (aOR 1.01, 95% CI 0.99–1.03) (Tables 2 and 3; Supplemental Fig. II). In the unadjusted analysis, thrombus density was associated with increased procedure duration (*β* 0.36, 95% CI 0.05–0.67 min longer per HU density increase) (*p* = 0.02). After adjustments, this association was not significant anymore (a*β* 0.27, 95% CI − 0.05–0.58 min longer per HU density increase) (Table 3; Supplemental Fig. III). Thrombus density was not associated with 24-h NIHSS (a*β* − 0.7, 95% CI − 1.4–0.2% change in NIHSS per HU increase). Only in the multivariable analysis thrombus density was associated with functional independence (aOR 1.02, 95% CI 1.00–1.05). The association between thrombus density and 90-day mRS is visualized in a mRS barplot (Fig. 1) and boxplot (Supplemental Fig. IV). No significant association was found between thrombus density and 90-day mortality (aOR 0.98, 95% CI 0.95–1.00) (Table 3).

**Table 3:** Association between thrombus density and outcomes

Outcome	Univariable analysis	Multivariable analysis
Final reperfusion grade (eTICI), median (IQR)^*^	cOR = 1.01 (0.99–1.03), *p* = 0.20	acOR = 1.01 (0.99–1.02), *p* = 0.48
First-pass reperfusion, *n* (%)^†^	OR = 1.01 (0.99–1.03), *p* = 0.29	aOR = 1.01 (0.99–1.03), *p* = 0.41
Procedure duration, median (IQR)^‡^	***β*** = 0.36 (0.05–0.67), ***p*** = 0.02	a*β* = 0.27 (− 0.05–0.58), *p* = 0.10
24-h NIHSS %, median (IQR)^‡^	*β* = 0 (− 0.9–0.9), *p* = 0.92	a*β* = − 0.7 (− 1.4–0.2), *p* = 0.09
Functional independence (mRS 0–2), *n* (%)^†^	OR = 1.01 (0.99–1.03), *p* = 0.32	aOR = 1.02 (1.00–1.05), *p = 0.05*
90-day mortality, *n* (%)^†^	OR = 0.99 (0.97–1.01), *p* = 0.29	aOR = 0.98 (0.95–1.00), *p* = 0.06

Multivariable analyses adjusted for age, sex, baseline NIHSS, time from onset to first CT imaging, intravenous alteplase treatment, distance from the terminus of the internal carotid artery (ICA-T) to thrombus (DT), clot burden score, Alberta stroke program early CT score (ASPECTS), and high-grade stenosis of ipsilateral carotid artery. eTICI 0 or 1 indicates no or minimal reperfusion, eTICI 2 indicates incomplete reperfusion (2A < 50% of territory; 2B ≥ 50% of territory, 2C near complete reperfusion except slow flow or a few small distal cortical emboli), and eTICI 3 indicates complete reperfusion

*eTICI* expanded thrombolysis in cerebral infarction, *IQR* interquartile range, *First-pass reperfusion* first-pass eTICI 2C-3, *NIHSS* National Institutes of Health Stroke Scale, *mRS* modified Rankin Scale, *(a)(c)OR* (adjusted) (common) odds ratio, *(a)β* (adjusted) beta coefficient, *CI* confidence interval

^*^Effect measure is the (a)cOR for ordinal outcomes (final reperfusion grade) for a 1-step shift towards better reperfusion or functional outcome per Hounsfield unit (HU) in thrombus density

^†^Effect measure is the (a)OR for dichotomous outcomes: first-pass reperfusion, functional independence, and 90-day mortality per HU increase in thrombus density. First-pass reperfusion was compared with multiple-pass reperfusion and no-excellent reperfusion (< 2C independent of the number of passes)

^‡^For the continuous outcomes of procedure duration and 24-h NIHSS, (a)*β* is displayed in the table. The (a)*β* for 24-h NIHSS indicates the percentage increase or decrease of 24-h NIHSS per HU, and for procedure duration, (a)*β* indicates increase or decrease in procedure duration in minutes per HU

No significant interaction was found between thrombus density and first-line treatment device for any of the outcomes. The interaction analysis results are reported in Supplemental Tables VI and VII along with an exploratory subgroup analysis.

## Discussion

In our study of 566 patients with M1 occlusions who were treated with EVT, thrombus density was not associated with final reperfusion grade, first-pass reperfusion, 24-h NIHSS, or mortality. Only in multivariable analysis, thrombus density was associated with functional independence. In patients with hyperdense thrombi, a stent retriever was used more often as first-line EVT device than in patients with non-hyperdense thrombi. However, we did not find significant interaction between thrombus density and first-line EVT device for any of the outcomes.

Contrary to some small studies [5–7], and a meta-analysis including five small studies [4], we did not find an association between thrombus density and reperfusion after EVT. However, heterogeneity of the meta-analysis was substantial (73%) [4]. Furthermore, occlusion location varied in all the aforementioned studies which might have affected the results because thrombus density differs significantly for different occlusion locations [11, 19]. Similar, in a previous study performed in patients with M1 occlusions only who underwent EVT [9], no association was found between thrombus density and reperfusion which is in line with our results.

Several studies described associations between thrombus density on NCCT and histological composition [3, 13–17]. Furthermore, fibrin-rich thrombi have been described to be more difficult to retrieve with EVT [3, 18]. So, one could have expected a relation between thrombus density and reperfusion outcomes. However, this association might have been affected by a change in thrombus composition with an increasing number of device passes. A previous study demonstrated that thrombus fragments retrieved during the first two passes are more RBC-rich than fragments retrieved in subsequent passes, the latter being more fibrin-rich [33]. These fibrin-rich portions of the thrombus might have contributed to the resistance to removal. Furthermore, this heterogeneity in thrombus composition might have been missed with our measurement method using only three ROIs to calculate average thrombus density [34]. Possibly, the non-hyperdense (fibrin-rich) parts of the thrombus were not captured with these three ROIs. In addition, it is important to keep in mind that the aforementioned study and most other histological studies simplify the complexity of thrombus composition by categorizing them in RBC-rich thrombi and fibrin-rich thrombi because these are the dominant components of thrombi retrieved in patients with acute ischemic stroke [16]. However, the role of platelets and their interaction with neutrophil extracellular traps (NETs) is not taken into account, while NETs have been associated with poor outcomes [35]. Thus, categorizing thrombi in RBC-rich, hyperdense thrombi and fibrin-rich, non-hyperdense thrombi does not take into account the more complex reality of the association between thrombus composition and thrombus density. Therefore, thrombus density measured on NCCT might fail to reflect the true complexity of thrombus composition.

It is possible that the inability to detect an association between thrombus density and reperfusion in our study was caused by a baseline imbalance in first-line EVT device between patients with hyperdense and non-hyperdense thrombi. However, this imbalance only existed in patients treated after 2016 because the use of an aspiration device became more common then. In addition, no significant interaction was found between thrombus density and first-line EVT device for any of the outcomes. Apparently, first-line device choice does not affect the association between thrombus density and (reperfusion) outcomes.

Contrary to other studies, thrombus density was significantly associated with functional independence in our study [8, 11]. However, this association was only significant in multivariable analysis, and the effect of the association was only small. In line with a previous study, we did not find an association between thrombus density and 90-day mortality [11]. Additionally, we investigated the association between thrombus density and 24-h NIHSS because this is a more direct measure of the effect of EVT and more sensitive than the mRS score at 3 months [36, 37]. Nonetheless, we were not able to detect an association between thrombus density and 24-h NIHSS.

Our study has limitations. First, thrombus measurements were performed by 10 trained observers which possibly caused interobserver variability. However, regular consensus readings with an expert neuroradiologist (CBLMM) were held to discuss difficult cases and compare measurements. Furthermore, a previous study demonstrated that non-expert observers could accurately and reproducibly assess thrombus density by the use of three ROIs [38]. Second, in our nationwide registry, reperfusion rates and functional outcome after EVT improved over the years, probably caused by improved workflow times and the increased experience of neuro-interventionists [39]. As a consequence, (reperfusion) outcomes of our study cohort included between March 2014 and November 2017 are probably worse than current clinical practice. As current reperfusion rates might be higher, the impact of a thrombus imaging characteristic such as thrombus density on (reperfusion) outcomes might be even smaller than reported in this study. Third, the main exclusion reason in our study was unavailability of thin-slice imaging which limited the total number of inclusions. Although acquired raw data are thin slices, in clinical practice, they are usually reconstructed into thicker slices to facilitate reading by reducing noise, and reduce the amount of data and processing times. Larger university hospitals more often store thin-slice imaging than smaller stroke centers. This may have led to an inclusion bias in our study cohort when compared to the overall cohort of M1 patients included in the MR CLEAN Registry. While this limited the generalizability of our results, it contributed to less scanner variability increasing the uniformity of our thrombus density measurements [40]. Finally, we used three ROIs to measure thrombus density which were placed in the proximal, middle, and distal part of the thrombus. A previous study found that full thrombus segmentations allow for more accurate thrombus density measurements [34]. Furthermore, with full thrombus segmentations, it is possible to detect heterogeneity of density values across the thrombus. Since we only used three ROIs, it might be possible that we missed these inhomogeneous components of the thrombus as mentioned before. Moreover, full thrombus segmentations can be automated and therefore are more likely to be implementable in clinical practice. As such, our results could support and stimulate future research on (automated) segmentation of the complete thrombus in order to account for heterogeneity of the thrombus and to further investigate the association between thrombus density, thrombus composition, and outcomes.

## Conclusion

In patients with acute ischemic stroke due to a M1 occlusion undergoing EVT, thrombus density was not clearly associated with final reperfusion grade, first-pass reperfusion, 24-h NIHSS, functional independence, and mortality. Therefore, no evidence was found that thrombus density is a useful predictor for EVT outcomes.

## Supplementary Information

Below is the link to the electronic supplementary material.

Supplementary file1 (PDF 623 KB)

## Data Availability

Source data of this study are not available due to privacy regulations, but analytic methods, and statistical code are available upon reasonable request.

## Abbreviations

EVT: Endovascular treatment
IVT: Intravenous alteplase treatment
eTICI: Extended thrombolysis in cerebral ischemia
mRS: Modified Rankin Scale
NIHSS: National Institutes of Health Stroke Scale
CTA: Computed tomography angiography
NCCT: Non-contrast computed tomography
HU: Hounsfield units
DT: Distance to thrombus
ASPECTS: Alberta Stroke Program Early CT Score
ICA-T: Terminus of internal carotid artery
RBC: Red blood cell
ENT: Embolization to a new territory
IQR: Interquartile ranges
a*β*: (Adjusted) *β* coefficient
acOR: (Adjusted) common odds ratio
aOR: (Adjusted) odds ratio

## Code availability

Statistical code is available upon reasonable request.
