# Supplementary material for: Association of thrombus density and endovascular treatment outcomes in patients with acute ischemic stroke due to M1 occlusions
Source: Neuroradiology. 2022 May 16;64(9):1857–67. doi: 10.1007/s00234-022-02971-4 (PMC9365751; doi:10.1007/s00234-022-02971-4)

Supplemental materials

Table of contents

Supplemental Tables

Supplemental Table I 3
Supplemental Table II 3
Supplemental Table III 4
Supplemental Table IV 5
Supplemental Table V 7
Supplemental Table VI 8
Supplemental Table VII 9

Supplemental Figures

Supplemental Figure I 10
Supplemental Figure II 11
Supplemental Figure III 12
Supplemental Figure IV 13

Supplemental Table I. Thrombus density across different scanner types

	Thrombus density in HU, median (IQR) (n=562)*	P value
**CT scanner model name**		0.08
Acquilion	55 (45-58), 15	
Acquilion One	51 (44-56), 57	
Acquilion PRIME	46 (42-52), 10	
Astelion	52 (52-52), 1	
BrightSpeed	54 (49-N/A), 3	
Brilliance 16	60 (N/A), 1	
Brilliance 40	47 (42-49), 5	
Brilliance 64	47 (43-55), 5	
Discovery CT	46 (42-N/A), 3	
Discovery CT750 HD	49 (44-52), 4	
iCT 256	48 (42-54), 124	
Ingenuity Core	50 (50-63), 6	
Ingenuity CT	47 (41-54), 6	
IQon - Spectral CT	47 (N/A), 1	
LightSpeed VCT	51 (48-54), 6	
Mx8000 IDT 16	53 (N/A), 1	
Optima CT660	49 (46-58), 11	
Revolution CT	37 (23-N/A), 2	
Sensation 16	53 (N/A), 1	
Sensation 64	50 (42-56), 55	
SOMATOM Definition AS	53 (45-60), 23	
SOMATOM Definition AS+	52 (46-61), 16	
SOMATOM Definition Edge	53 (45-59), 54	
SOMATOM Definition Flash	51 (45-58), 108	
SOMATOM Force	55 (47-61), 44	

HU: Hounsfield Units. IQR: interquartile range. *Number of patients with available CT scanner model name

Supplemental Table II. First-line treatment device over the years

	**2014 (n=43)**	**2015 (n=139)**	**2016 (n=160)**	**2017 (n=224)**	**P value**
**First-line EVT device, n/total n, (%)**					**<0.001**
Stent-retriever	33/39 (85)	86/107 (80)	97/118 (82)	106/183 (58)*	
Aspiration	6/39 (15)	21/107 (20)	21/118 (18)	77/183 (42)	

EVT: endovascular treatment.

Supplemental Table III. First-line treatment device in patients with hyperdense vs non-hyperdense thrombi included in 2014-2016 vs 2017

	**2014-2016 (n=342)**		**P value**	**2017 (n=224)**		**P value**
	Hyperdense thrombi (n=190)	Non-hyperdense thrombi (n=152)	0.2	Hyperdense thrombi (n=109)	Non-hyperdense thrombi (n=115)	**0.01**
**First-line EVT device, n/total n, (%)**						
Stent-retriever	124/147 (84)	92/117 (79)		58/86 (67)	48/97 (49.5)	
Aspiration	23/147 (16)	25/117 (21)		28/86 (33)	49/97 (50.5)	

EVT: endovascular treatment; hyperdense thrombi: thrombi ≥50 Hounsfield Units (HU); non-hyperdense thrombi: thrombi <50 HU.

Supplemental Table IV. Baseline characteristics of patients with M1 occlusions included in our study and of the complete group of patients with M1 occlusions included in the MR CLEAN Registry.

**Baseline characteristics**	**Subgroup M1 patients (n=566)**	**All M1 patients (n=1815)**	**P value**
Age, median (IQR), total n	72 (62-80), 566	73 (62-81), 1815	0.78
Male, n/total n (%)	285/566 (50)	908/1815 (50)	0.85
**Medical History, n/total n (%)**			
Diabetes	87/560 (16)	297/1806 (16)	0.49
Hypertension	252/552 (46)	942/1779 (53)	**<0.001**
Hypercholesterolemia	137/540 (25)	534/1730 (31)	**0.001**
Previous stroke	93/561 (17)	316/1804 (18)	0.48
Myocardial infarction	75/555 (14)	272/1787 (15)	0.18
Atrial fibrillation	145/557 (26)	450/1791 (25)	0.55
Anticoagulation use (vitamin K antagonists)	79/562 (14	227/1801 (13)	0.21
Direct anticoagulants	24/560 (4)	65/1796 (4)	0.31
**Clinical presentation**			
Baseline NIHSS, median (IQR), total n	16 (11-20), 559	16 (11-19), 1789	0.55
Prestroke mRS, n/total n (%)			0.69
0-2	486/556 (87)	1565/1781 (88)	
≥3	70/556 (13)	216/1781 (12)	
**Imaging characteristics**			
Baseline ASPECTS score, median (IQR), total n	9 (8-10), 566	9 (8-10), 1781	0.41
Clot burden score, median (IQR), total n	6 (6-7), 447	6 (6-7), 1479	0.58
Thrombus absolute density in HU, median (IQR), total n†	50 (44-57), 566	N/A	-
Distance from ICA-T to thrombus (DT) in mm, median (IQR), total n†	13 (6-18),566	N/A	-
**Collaterals, n/total n (%)**			**0.03**
0. Absent collaterals	35/557 (6)	95/1766 (5)	
1. ≤50% filling of the occluded territory	206/557 (37)	623/1766 (35)	
2. >50% and <100% filling of the occluded territory	221/557 (40)	696/1766 (39)	
3. 100% filling of the occluded territory	95/557 (17)	352/1766 (20)	
Ipsilateral atherosclerotic carotid artery stenosis >50%, n/total n (%)	43/506 (9)	161/1642 (10)	0.24
Ipsilateral total atherosclerotic occlusion of carotid artery, n/total n (%)	39/506 (8)	117/1642 (7)	0.54
Ipsilateral dissection of carotid artery, n/total n (%)	7/506 (1)	27/1642 (2)	0.58
IVT prior to EVT, n/total n (%)	413/565 (73)	1366/1818 (75)	0.10
Onset to first CT imaging in min, median (IQR), total n ‡	74 (54-120), 406	73 (53-121), 1284	0.66

Transfer patient, n/total n (%)	212/565 (38)	1014/1814 (56)	**<0.001**
University hospital, n/total n (%)	372/566 (66)	1022/1818 (56)	**<0.001**
Onset to groin time in min, median (IQR), total n	185 (138-245), 561	195 (150-255), 1807	**<0.001**

Categorical variables are presented as n/N and percentage. Continuous variables are presented as median (interquartile range [IQR]), total number. NIHSS: National Institutes of Health Stroke Scale; mRS: modified Rankin Scale; M1: M1 segment of the middle cerebral artery; ASPECTS: Alberta stroke program early CT score; ICA-T: terminus of internal carotid artery; IVT: intravenous alteplase treatment**;** EVT: endovascular treatment; Transfer patient: patients who initially presented at a primary stroke center and were transferred to a comprehensive stroke center for EVT; University hospital: number of EVT procedures performed in university hospitals † Thrombus measurements were performed on thin slice non-contrast CT (NCCT) and CT angiography (CTA) imaging. Median slice thickness of NCCT was 1 mm (IQR, 0.9-1 mm) and median slice thickness of CTA was 0.75 mm (IQR, 0.6-1 mm); [‡] First CT imaging refers to the admission imaging used for thrombus imaging characteristics.

Supplemental Table V. Comparison of CT scanner models in patients with M1 occlusions included in our study and the complete group of patients with M1 occlusions included in the MR CLEAN Registry

	Subgroup M1 patients (n=562)*	All M1 patients (n=1590)*	P value
**CT scanner model name, n/total n (%)**			**<0.001**
Acquilion	15/562 (3)	80/1590 (5)	
Acquilion One	57/562 (10)	126/1590 (8)	
Acquilion PRIME	10/562 (2)	24/1590 (2)	
Astelion	1/562 (0.2)	1/1590 (0.06)	
Biograph 40	0/562 (0)	1/1590 (0.06)	
BrightSpeed	3/562 (0.5)	3/1590 (0.2)	
BrightSpeed S	0/562 (0)	74/1590 (5)	
Brilliance 16	1/562 (0.2)	13/1590 (0.8)	
Brilliance 40	5/562 (0.9)	25/1590 (2)	
Brilliance 64	5/562 (0.9)	141/1590 (9)	
Discovery CT	3/562 (0.5)	10/1590 (0.6)	
Discovery CT750 HD	4/562 (0.7)	35/1590 (2)	
Emotion 16 (2007)	0/562 (0)	3/1590 (0.2)	
Emotion 16 (2010)	0/562 (0)	1/1590 (0.06)	
iCT 256	124/562 (22)	278/1590 (17)	
Ingenuity Core	6/562 (1.1)	14/1590 (0.9)	
Ingenuity CT	6/562(1.1)	14/1590 (0.9)	
IQon - Spectral CT	1/562 (0.2)	8/1590 (0.5)	
LightSpeed VCT	6/562(1.1)	21/1590 (1.3)	
LightSpeed16	0/562 (0)	1/1590 (0.06)	
Mx8000 IDT 16	1/562 (0.2)	4/1590 (0.2)	
Optima CT660	11/562 (2)	34/1590 (2)	
Revolution CT	2/562 (0.4)	2/1590 (0.1)	
Sensation 16	1/562 (0.2)	8/1590 (0.5)	
Sensation 16 with Akron Q tube	0 /562 (0)	1/1590 (0.06)	
Sensation 64	55/562 (10)	95/1590 (6)	
SOMATOM Definition AS	23/562 (4)	169/1590 (11)	
SOMATOM Definition AS+	16/562 (3)	45/1590 (3)	
SOMATOM Definition Edge	54/562 (10)	90/1590 (6)	
SOMATOM Definition Flash	108/562 (19)	210/1590 (13)	
SOMATOM Force	44/562 (8)	59/1590 (4)	
**Total number of CT models used**	25	31	

M1: M1 segment of the middle cerebral artery *Number of patients with available CT scanner model name

Supplemental Table VI. Unadjusted effects of thrombus density on first-line stent retriever and first-line aspiration endovascular treatment

Outcome (c)OR/β and 95% CI for thrombus density	Interaction p=	First-line stent-retriever (n=322)§	First-line aspiration (n=125)§
Final reperfusion grade (eTICI), median (IQR)*	0.75	cOR = 1.02 (1.00-1.04), p=0.12	cOR = 1.00 (0.97-1.03), p=0.79
First-pass reperfusion, n (%)†	0.53	OR = 1.01 (0.99-1.04), p=0.37	OR = 1.01 (0.97-1.05), p=0.73
Procedure duration, median (IQR) ‡	0.86	β = 0.36 (-0.04-0.8), p=0.08	β = 0.03 (-0.6-0.6), p=0.93
24-hour NIHSS %, median (IQR) ‡	0.26	β = 0.9 (-0.2-1.9), p=0.17	β = -1.6 (-3.4-0.2), p=0.09
Functional independence (mRS 0-2), n (%)†	0.33	OR = 1.00 (0.97-1.02), p=0.74	OR = 1.04 (0.99-1.08), p=0.10
90-day mortality, n (%) †	0.51	OR = 1.00 (0.97-1.03), p=0.96	OR = 0.97 (0.93-1.01), p=0.17

Interaction: effect of thrombus density on first-line treatment device was assessed by adding an interaction term (first-line treatment device*thrombus density) to the regression model; eTICI: expanded thrombolysis in cerebral infarction. eTICI 0 or 1 indicates no or minimal reperfusion, eTICI 2 indicates incomplete reperfusion (2A: <50% of territory; 2B: ≥50% of territory, 2C: near complete reperfusion except slow flow or a few small distal cortical emboli), and eTICI 3 indicates complete reperfusion; IQR: interquartile range; First-pass reperfusion: First-pass eTICI 2C-3; NIHSS: National Institutes of Health Stroke Scale; mRS: modified Rankin Scale; (c)OR: (common) odds ratio; β: beta coefficient; CI: confidence interval. * Effect measure is the cOR for ordinal outcomes (final reperfusion grade) for a 1-step shift towards better reperfusion per Hounsfield Unit (HU) in thrombus density. † Effect measure is the OR for dichotomous outcomes: first-pass reperfusion, functional independence and 90-day mortality per HU increase in thrombus density. First-pass reperfusion was compared with multiple-pass reperfusion and no-excellent reperfusion (<2C independent of the number of passes). ‡ For the continuous outcomes of procedure duration and 24-hour NIHSS, β are displayed in the table. The β for 24-hour NIHSS indicates the percentage increase or decrease of 24-hour NIHSS per HU and for procedure duration β indicates increase or decrease in procedure duration in minutes per HU. § Number of patients with first-line treatment device recorded (n=447).

Supplemental Table VII. Adjusted effects of thrombus density on first-line stent retriever and first-line aspiration endovascular treatment

**Outcome a(c)OR/ aβ and 95% CI for thrombus density¶**	**Interaction p=**	**First-line stent-retriever (n=322)§**	**First-line aspiration(n=125)§**
Final reperfusion grade (eTICI), median (IQR)*	0.75	acOR = 1.02 (0.99-1.04), p=0.14	acOR = 0.99 (0.95-1.02), p=0.51
First-pass reperfusion, n (%)†	0.56	aOR = 1.02 (0.99-1.05), p=0.29	aOR = 1.00 (0.96-1.05), p=0.98
Procedure duration, median (IQR)‡	0.89	aβ = 0.24 (-0.2-0.7), p=0.25	aβ = 0.003 (-0.6-0.6), p=0.99
24-hour NIHSS %, median (IQR)‡	0.25	aβ = -0.2 (-1.1-0.9), p=0.76	aβ = -1.4 (-2.9-0.2), p=0.10
Functional independence (mRS 0-2), n (%)†	0.43	aOR = 1.02 (0.98-1.05), p=0.34	aOR = 1.03 (0.98-1.08), p=0.33
90-day mortality, n (%)†	0.80	aOR = 0.98 (0.94-1.02), p=0.25	aOR = 0.97 (0.92-1.02), p=0.26

Interaction: effect of thrombus density on first-line treatment device was assessed by adding an interaction term (first-line treatment device*thrombus density) to the regression model; eTICI: expanded thrombolysis in cerebral infarction. eTICI 0 or 1 indicates no or minimal reperfusion, eTICI 2 indicates incomplete reperfusion (2A: <50% of territory; 2B: ≥50% of territory, 2C: near complete reperfusion except slow flow or a few small distal cortical emboli), and eTICI 3 indicates complete reperfusion; IQR: interquartile range; First-pass reperfusion: First-pass eTICI ≥2C; NIHSS: National Institutes of Health Stroke Scale; mRS: modified Rankin Scale; a(c)OR: adjusted (common) odds ratio; aβ: adjusted beta coefficient; CI: confidence interval. * Effect measure is the acOR for ordinal outcomes (final reperfusion grade) for a 1-step shift towards better reperfusion or functional outcome per Hounsfield Unit (HU) in thrombus density. † Effect measure is the aOR for dichotomous outcomes: first-pass reperfusion, functional independence and 90-day mortality per HU increase in thrombus density. First-pass reperfusion was compared with multiple-pass reperfusion and no-excellent reperfusion (<2C independent of the number of passes). ‡ For the continuous outcomes of procedure duration and 24-hour NIHSS, aβ are displayed in the table. The aβ for 24-hour NIHSS indicates the percentage increase or decrease of 24-hour NIHSS per HU and for procedure duration aβ indicates increase or decrease in procedure duration in minutes per HU. § Number of patients with first-line treatment device recorded (n=447). ¶ Adjustments: age, sex, baseline NIHSS, time from onset to first CT imaging, intravenous alteplase treatment, distance from the terminus of the internal carotid artery (ICA-T) to thrombus (DT), clot burden score, Alberta stroke program early CT score (ASPECTS), high grade stenosis of ipsilateral carotid artery.

Supplemental Figure I. Flowchart of the patient selection procedure. *Movement, metal and beam hardening artefacts

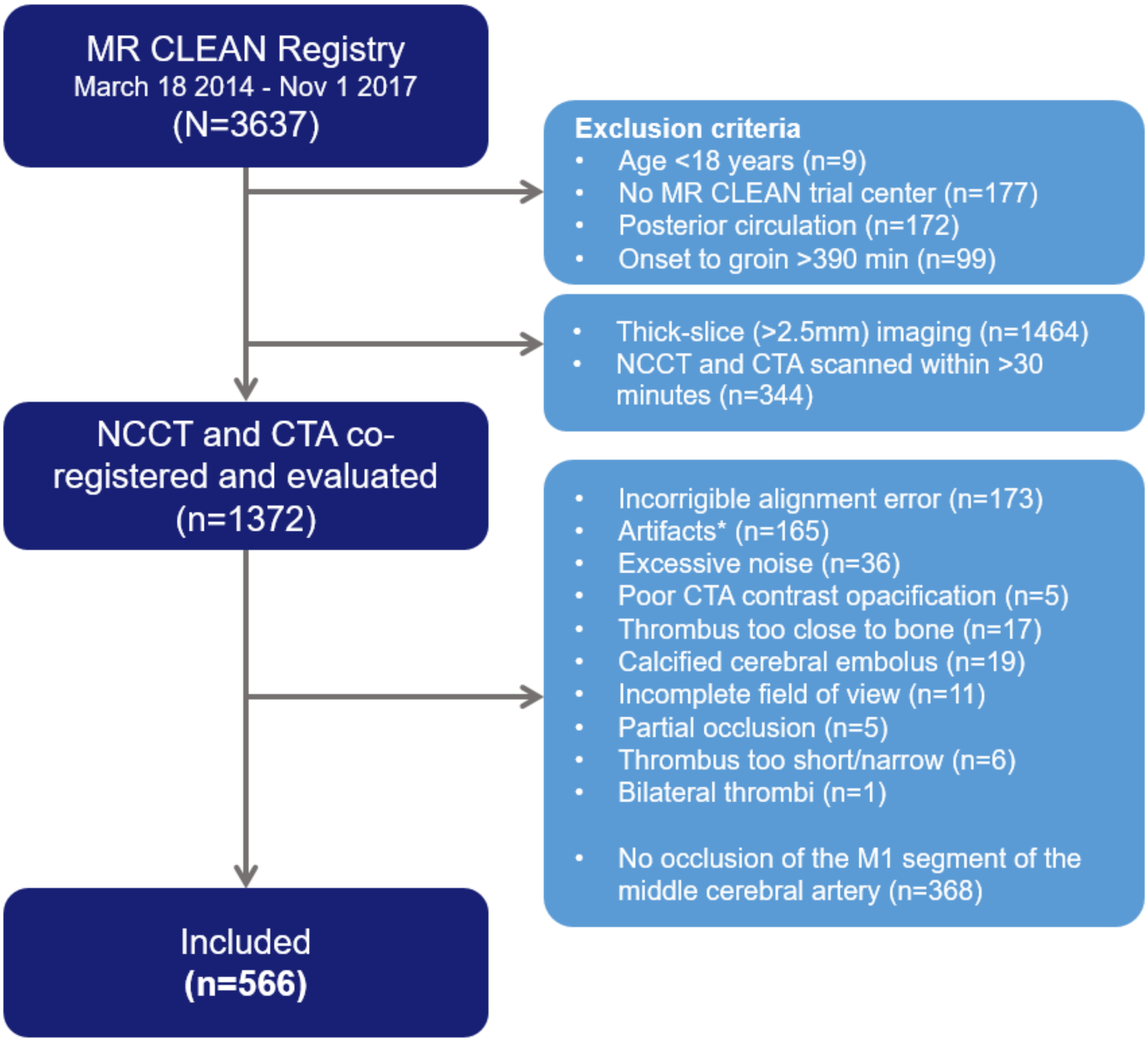

Supplemental Figure II. Distribution of thrombus density in Hounsfield Units (HU) per expanded thrombolysis in cerebral infarction (eTICI) score. eTICI 0 or 1 indicates no reperfusion or minimal reperfusion. eTICI 2 indicates incomplete reperfusion (2A: <50% of territory; 2B: ≥50% of territory, 2C: near complete reperfusion except slow flow or a few small distal cortical emboli). eTICI 3 indicates complete reperfusion.

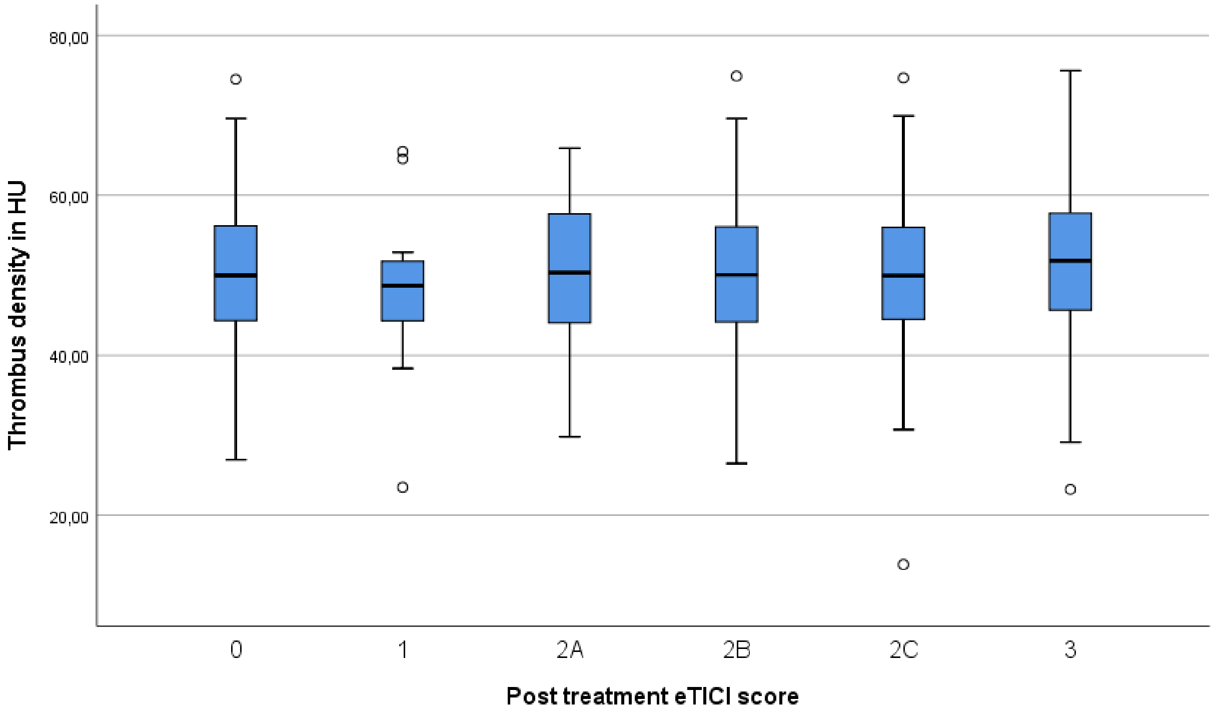

Supplemental Figure III. Distribution of thrombus density in Hounsfield Units (HU) for duration of endovascular treatment (EVT) procedure in minutes.

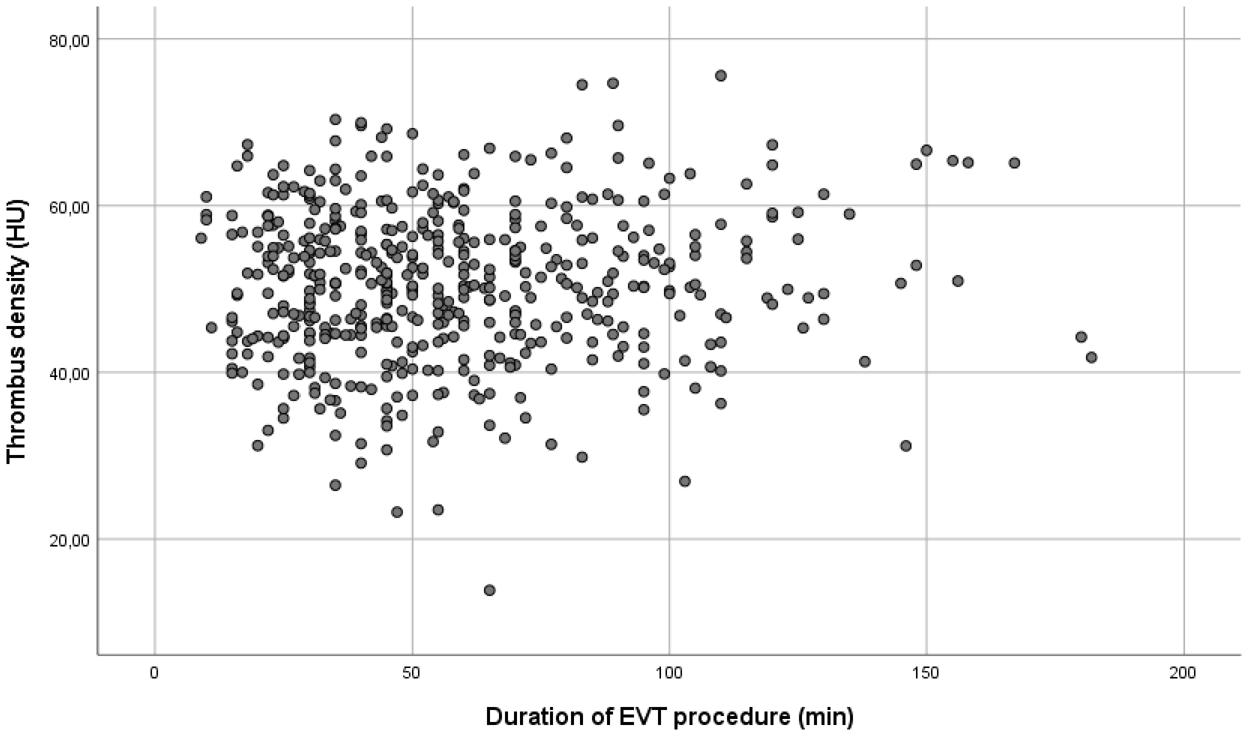

Supplemental Figure IV. Distribution of thrombus density in Hounsfield Units (HU) per 90-day modified Rankin Scale (mRS) score.

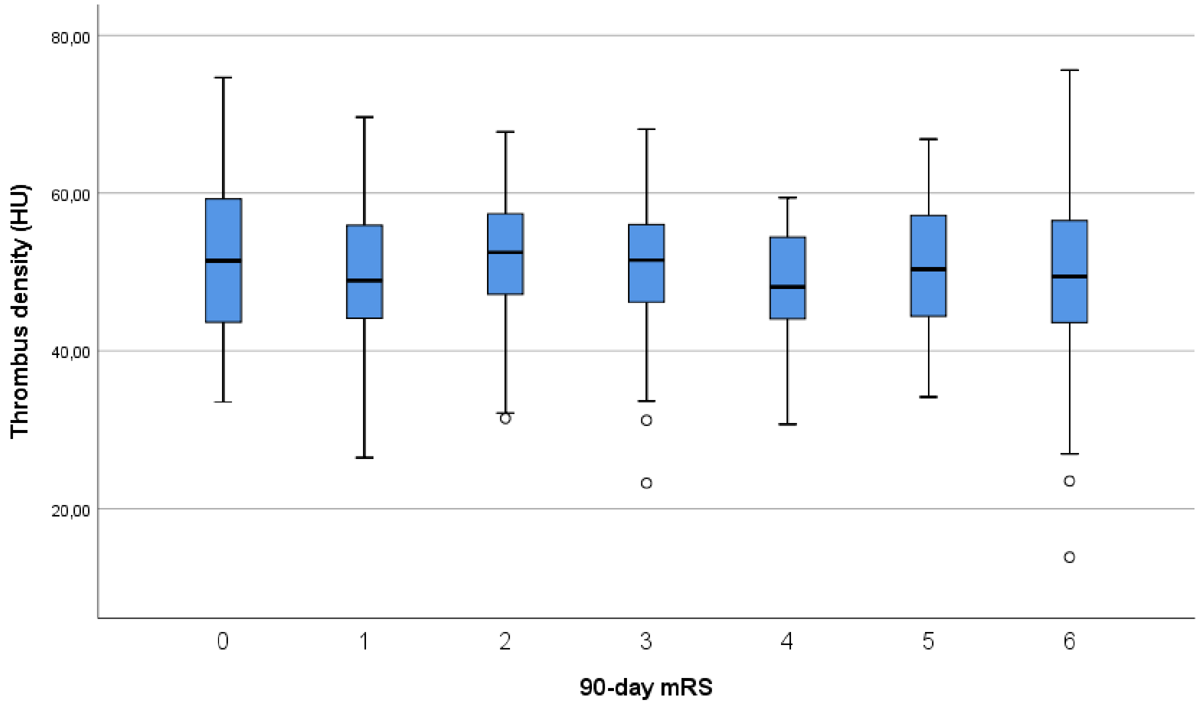

Supplement: Supplementary file 1 — Supplementary file1 (PDF 623 KB) [file 234_2022_2971_MOESM1_ESM.pdf]
